# Toward a New Future for Essential Oils

**DOI:** 10.3390/antibiotics10020207

**Published:** 2021-02-19

**Authors:** Edoardo Napoli, Maura Di Vito

**Affiliations:** 1 Istituto Chimica Biomolecolare—C.N.R., Via Paolo Gaifami 18, 95126 Catania, Italy; 2 Dipartimento di Scienze e Tecnologie Agro-Alimentari, Università of Bologna, Viale G. Fanin 42, 40127 Bologna, Italy; 3 Dipartimento di Scienze Biotecnologiche di Base, Cliniche Intensivologiche e Perioperatorie, Università Cattolica del Sacro Cuore, Largo A. Gemelli 8, 00168 Rome, Italy

Essential oils (EOs) are peculiar phytocomplexes in the already widely varied world of natural bioactive substances. They represent the volatile and aromatic components of some officinal plants, called aromatic plants [1], obtained through well-regulated extraction techniques [2]. They have been known, used, and studied for therapeutic purposes since ancient times, when, due to their evocative power, they were also considered a means of communication between humans and spiritual entities. However, it is their uses as therapeutic agents in the form of active ingredients belonging to the spices and to preserve food that have driven increasing interest in their use over the years. Their fame as antiseptic substances derive from their use in traditional medicines around the world [3].

Antibiotic multi-resistance has become a global emergency in recent decades. The indiscriminate use of antibiotic substances in both human and veterinary fields has led us from the dream of magic pills to the modern nightmare of the ineffectiveness of many previously active molecules. Despite all efforts, international research has struggled to find new and effective chemical drugs, so is looking to the natural world as a source of new scaffolds with new antibiotic activity. The broad-spectrum antimicrobial activity manifested by EOs, in a similar context, must be noticed. Their nature as a complex chemical mixture, which varies in terms of the quantity of their individual bioactive components, makes them resistant to any mechanism of microbial resistance. However, much remains to be understood about their mechanism of action, although often it is attributed to their ability to interact with the bacterial microbial wall [4]. The control of microbial growth is also an important problem for the food industry (food deterioration and shelf-life extension) and for cultural heritage artworks (indoor and outdoor biodeterioration) [5,6,7,8]. In this scenario, EOs can play an important role.

EOs have also shown antiviral activity, a peculiarity that is arousing some interest, especially in recent years [9,10]. The use of these natural compounds for inhalation and their interactions with the central nervous system are still topics of fascination that, however, require more robust scientific confirmation, which we expect in the near future due to the numerous research groups that are lending their attention in this field [11,12,13].

However, is there another side to this coin? Despite their considerable application potential, EOs present a series of problems that have limited their use on a large scale to date.

The first obstacle to the widespread diffusion of EOs is their potential for acute and chronic toxicity and their role in the development of some allergic reactions. Although most of the components considered individually and many EOs in their entirety are generally recognized as safe (GRAS), their use is often conditioned by restrictions that are not supported by adequate scientific documentation or are based on literature data that are not yet sufficient to determine the correct exposure doses. Therefore, their use is avoided according to the prudence principle. Additional effort by the scientific community in this regard is highly desirable.

Another problem that is important is their chemical variability. Being composed of secondary metabolites, they are considerably affected by external factors (climatic, agronomic, and anthropic), which make them extremely qualitatively and quantitatively variable over time. This problem has been, in part, limited due to the introduction of the chemotyping of EOs. An effort to fix the chemical compositions of EOs through analysis of large plant populations as well as the search for new endemics and new chemotypes must not fail to consider the variability derived from the biodiversity of the various aromatic species.

The extremely lipophilic nature, volatility, and susceptibility to oxidation shown by EOs also represent a limitation, especially technological, to their application. The lipophilicity makes them hardly usable in polar solvents, whereas their volatility and instability in the air has made their application difficult, especially in the agronomic field. The many studies on the applicability of nanotechnologies to overcome these limits has opened new perspectives that, until now, we could not have imagined.

Hydrolates (Hys), hydrophilic co-products of EOs, are increasingly attracting the interest of the scientific community due to their versatile characteristics. Although more perishable than EOs, these natural compounds have fair antimicrobial activity, are generally safe, and do not need to be diluted in a vehicle before use. These characteristics make them suitable not only for human and veterinary medicine (for both topical applications and oral administration), but also for other applications such as environmental, entomological, and agronomic applications [14,15,16].

In this Special Issue, we selected 13 manuscripts from eminent research groups, each of which provides a significant advance in knowledge about the biological activities and potential applications of EOs. Seven of them deal with antibacterial activity with reference to human, veterinary, and food applications. The potential antibacterial and antibiofilm activities of *Pimenta dioica* and *Pimenta racemosa* EOs in relation to their chemical composition, in addition to their ability to treat *Acinetobacter baumannii* wound infection in a mouse model, were investigated by Ismail et al. [17]. *P. dioica* leaf EO efficiently inhibited and eradicated biofilm formed by *A. baumannii*. Both *P. dioica* and *P. racemosa* leaf EOs also showed bactericidal activity. In addition, a significant reduction in *A. baumannii* microbial load in a mouse wound infection model was found.

Recent evidence suggests that *Staphylococcus* spp. has the ability to colonize the reproductive system and to affect its structure and functions. Kačániová et al. [18] focused on the antibacterial effects of 14 selected EOs against 50 *Staphylococcus* spp. cultures isolated from human semen. The best anti-*Staphylococcus* activities were found for the EOs of *Canarium luzonicum* (Blume) A. Gray, *Amyris balsamifera*, *Cinnamomum camphora*, and *Pogostemon cablin*.

Di Vito et al. [19] studied the in vitro antimicrobial and anti-biofilm effectiveness of both *Origanum vulgare* EO and a commercial product based on EOs on 29 *Salmonella* spp. strains isolated from chicken and pig farms. The authors concluded on the possible use of a commercial formulation both to reduce the meat contamination of *Salmonella* spp. before slaughter and in synergy with low doses of ciprofloxacin against resistant strains diffusion.

Di Stefano et al. [20] focused on the comparative study of the antimicrobial and antifungal activities of different grades of EOs extracted from the resins of three different *Boswellia sacra* cultivars toward relevant pathogens. One EO showed a minimum inhibitory concentration (MIC) of 52 mg/mL toward both *Staphylococcus aureus* and *Pseudomonas aeruginosa* pathogens, while other samples were particularly active against a dermatological strain of *Propionibacterium acnes*. Data obtained from in vitro studies showed that all EOs had a significant antifungal effect against *Candida albicans* and *Malassezia furfur*.

The biological activities of an EO distilled by Brazilian medicinal plant *Aniba rosaeodora* were evaluated by Teles et al. [21]. This EO, with linalool as its major compound, showed activity against all the bacteria strains tested, standard strains, and marine environment bacteria, with the lower minimum inhibitory concentration observed for *S. aureus*. The antitrypanosomal activity of *A. rosaeodora* EO and linalool was observed at high concentrations against epimastigote forms and even higher concentrations against intracellular amastigotes of *Trypanosoma cruzi*. Both *A. rosaeodora* EO and linalool did not exhibit a cytotoxic effect in BALB/c peritoneal macrophages, and both reduced nitrite levels in unstimulated cells, revealing a potential effect on nitric oxide production.

The inhibition of glucosyltransferase activity and glucan production as an antibiofilm mechanism of lemongrass EO against *Escherichia coli* O157:H7 was studied by Ortega-Ramirez et al. [22]. The planktonic growth of *E. coli* was inhibited by EO, citral, and geraniol as per as the bacterial adhesion on stainless steel. All compounds decreased the glucans production. The evidence collected by docking analysis indicated that both terpenes could interact with the helix finger of the glucosyltransferase responsible for polymer production.

Finally, the antimicrobial activity of some hydrolates was evaluated in comparison with the corresponding EOs by Di Vito et al. [23]. The authors highlight that although the minimum inhibitory concentration values of the EOs are lower than the corresponding Hys, the volatiles contained in the Hys are more effective in inhibiting microbial growth because they are active at lower concentrations. These data support the growing interest in Hys, especially when it is necessary to act in hydrophilic environments with products safer than essential oils.

The antifungal potential of EOs was investigated in three studies. An example of the use of EOs as novel alternatives to the application of synthetic fungicides to control seedborne pathogens is provided by Moumni et al. [24], with their in vitro study conducted on the growth inhibition of seven EOs against *Stagonosporopsis cucurbitacearum* and *Alternaria alternata.* EO with citral, β-myrcene, and geraniol as major components controlled these fungi most effectively, followed by EOs containing terpinen-4-ol or linalool. Kisova et al., using a microarray approach, provide an important contribution to understanding the mechanism of action of EOs against fungi [25]. They evaluated the gene expression of *Penicillium chrysogenum* exposed to the indirect contact (vapors) of eight EOs. A microarray investigation confirmed their main impact on the metabolic processes in *P. rubens* involved vital functions.

Nanoencapsulation of EOs is a promising topic in nanotechnology. Ecofriendly EOs nanosuspensions with their broad-spectrum antimicrobial activity could be a valid alternative to synthetic products. The antifungal activity of a nanoencapsulated EO of oregano and thyme was evaluated by Kapustova et al. [26]. Their results showed that the nanosystems containing both thyme and oregano EOs are active against various fungal strains belonging to natural environments and materials. In particular, the minimum inhibitory concentration and minimum fungicidal concentration values were two to four times lower than EOs alone. This result suggests interesting applications in the agri-food and environmental fields.

Two studies were devoted to the use of EOs as chemotaxonomic markers. This is an approach that finds application both in botany, providing a tool for the enhancement of biodiversity and endemics, and in the quality control of EOs. The differences in the composition of EOs obtained from the aerial parts of six *Ferula* species, determined by also using a chemometric approach, were addressed by Youssef et al. [27]. In this manuscript, the authors evaluate the in vitro antioxidant potential of the EOs together with tyrosinase inhibitory potential using different assays, concluding that *Ferula* species could serve as a promising natural antioxidant drug that could be included in different cosmetics or pharmaceutical products to counteract hyperpigmentation.

The results of gas chromatographic analytical techniques coupled with random amplified polymorphic DNA (RAPD) techniques allowed Kim et al. [28] to obtain a perfect distinction between two Korean thyme cultivars (Wolchul and Odae) and commercial thyme cultivars.

The role of EOs as feeding agents for farm animals and their effect on meat quality is a topic of interest. The beneficial effect of oregano EO administration on animal diet was demonstrated by Garcia-Galicia et al. [29] in a study on lambs. This work demonstrates that oregano EO was beneficial for lambs feeding and could be a natural alternative to replace monensin in lamb diets, with improvements demonstrated in the quality of the meat.

In conclusion, the works published in this Special Issue provide a broad and clearly non-exhaustive vision of the application potential of EOs and show the path that is being followed for their application in the food, human health, veterinary fields, with particular reference to the control of microbial and fungal infections and/or contaminations. We expect this Special Issue to be of great help to those interested in the valorization and exploitation of EOs.
